# 

**Published:** 1890-05

**Authors:** 


### TABLE OF CONTENTS.

ORIGINAL COMMUNICATIONS. PAGE

The Dental Crown and its Enabling Method of Removable Bridge-Work. By William H. Gates, D.D.S., Philadelphia . . . . 257

Correcting Irregularities by the Spring of Gold Bands. By B. S. Byrnes, D.D.S., Memphis, Tenn. . . . . 263

Some Practical Points learned at Society Meetings and Clinics. By B. A. R. Ottolengui, New York City . . . . 267

REPORTS OF SOCIETY MEETINGS.

Monthly Meeting of the American Academy of Dental Science, held at the Boston Medical Library Association Rooms, February 5, 1890 . . . . 274

Seventh Annual Session of the Maryland State Dental Association . . . . 286

Odontological Society of Pennsylvania . . . . 296

Union Meeting of the Connecticut Valley Dental Society, the New England Dental Society, and the Connecticut State Dental Association, at Springfield, Mass., October 23, 24, and 25, 1889 . . . . 301

EDITORIAL.

Dental Societies . . . . 310

Bibliography . . . . 313

FOREIGN CORRESPONDENCE . . . . 314

DOMESTIC CORRESPONDENCE . . . . 315

CURRENT NEWS . . . . 318

